# Determining Barriers to Submitting Antimicrobial-Resistant Isolates to the Texas Department of State Health Services (DSHS) Antimicrobial Resistance Laboratory Network (AR Lab Network): A Cross-Sectional Study

**DOI:** 10.7759/cureus.109758

**Published:** 2026-05-27

**Authors:** Ryan Nguyen, Rachel Pittman, Rebecca L Sanchez

**Affiliations:** 1 Department of Applied Biomedical Sciences, University of the Incarnate Word School of Osteopathic Medicine, San Antonio, USA

**Keywords:** antimicrobial stewardship (ams), infectious disease control, multidrug-resistant pathogen, primary survey, public health surveillance

## Abstract

Background: The Antimicrobial Resistance Laboratory Network (AR Lab Network) was developed by the Centers for Disease Control and Prevention (CDC) to detect and prevent antimicrobial-resistant threats. However, low submission rates of antimicrobial-resistant isolates limit the AR Lab Network’s ability to address antimicrobial resistance (AMR). This study expands on a study conducted in Texas Public Health Region 8 (PHR8).

Aim: The aim of this study was to investigate submission barriers for antimicrobial-resistant isolates in Texas acute care hospitals (ACH) and critical access hospitals (CAH).

Methods: A survey was designed and emailed to laboratory professionals to identify barriers to antimicrobial-resistant isolate submission. Responses were analyzed using two-sided Fisher’s exact tests to identify associations between responses and respondent characteristics.

Results: Of the Texas laboratory personnel invited to participate, 123 responses from 211 hospitals were received, for a response rate of 58.29%. Lack of awareness of the AR Lab Network was the most frequently cited submission barrier (50.48% of respondents). Other submission barriers included submission to another laboratory (49.53%), lack of staff time (42.86%), lack of training or certified personnel (41.9%), and a submission process that was too time-consuming (40%).

Discussion: As in the Texas PHR8 study, it was found that, regardless of the respondent’s role, time in that role, or the type of hospital in which they worked, the most common barrier to antimicrobial-resistant isolate submission was lack of awareness of the AR Lab Network. In the future, the identified barriers will be addressed by implementing educational outreach programs about the AR Lab Network for Texas hospitals and healthcare facilities.

## Introduction

According to the Centers for Disease Control and Prevention (CDC) 2019 Antibiotic Resistance Report in the United States, more than 2.8 million antimicrobial-resistant infections occur each year, resulting in more than 35,000 deaths. Using data to detect and track resistance is one of the five core actions identified by the CDC to help the United States combat the threat of antimicrobial resistance (AMR) [1]. Tracking local AMR data helps public health departments identify where AMR is on the rise and inform outbreak responses. In 2016, the CDC created the Antimicrobial Resistance Laboratory Network (AR Lab Network) to expand laboratory infrastructure and capacity, with state and local public health laboratories testing for antimicrobial-resistant organisms using CDC guidelines and Clinical Laboratory Improvement Amendments (CLIA) requirements. The AR Lab Network has increased testing capacity in all 50 states, five cities, and Puerto Rico, allowing for a faster response to outbreaks, and has also created a platform for communication with clinical and public health laboratory partners, expediting the collection of AMR data across various healthcare facilities.

Since June 2017, the Texas Department of State Health Services (DSHS) AR Laboratory has made significant contributions to the AR Lab Network by testing thousands of submitted isolates of novel or targeted antimicrobial-resistant organisms in Texas. The AR Lab Network targeted organisms in Texas include, but are not limited to, carbapenem-resistant Enterobacterales (CRE), carbapenem-resistant Pseudomonas aeruginosa (CRPa), carbapenem-resistant Acinetobacter baumannii (CRAb), and Candida auris or Candida species that are unable to be identified by the testing facility. The Texas DSHS AR Laboratory provides testing for organism identification, antimicrobial susceptibility, carbapenemase production, and mechanism of resistance [2]. Although isolate submission to the AR Lab Network is voluntary in Texas, DSHS has recruited facilities to submit isolates to the Texas DSHS AR Laboratory through mailed recruitment letters, word of mouth, and promotion at infection prevention meetings. In addition to encouraging participation in the CDC AR Lab Network, DSHS provides testing for healthcare facilities in Texas without the resources to test their own isolates and submits data from these samples to the AR Lab Network. Additionally, isolates are requested by Healthcare-Associated Infections (HAI) epidemiologists while conducting epidemiological investigations to contain and prevent the spread of AMR in healthcare facilities. However, DSHS reports that only 4% of facilities statewide have historically submitted isolates, limiting the potential of the AR Lab Network to address AMR in Texas. For example, between 2019 and 2021, there were 217 CRE cases reported to DSHS in Public Health Region 8 (PHR8), a region of Texas comprising 28 counties [3], but only 86 CRE isolates were tested by the AR Lab Network. Our group previously reported on barriers to antimicrobial-resistant isolate submission by acute care hospitals (ACH) and critical access hospitals (CAH) in PHR8. In that study, it was found that the primary barrier to isolate submission was a lack of awareness of the AR Lab Network, followed by a lack of laboratory staff time and training [4]. The aim of this study was to expand upon our previous study to determine whether the barriers to isolate submission experienced by ACH and CAH in Texas PHR8 are also present in these facilities throughout Texas. Identifying barriers to submission and working to eliminate them may increase isolate submissions and ultimately allow Texas DSHS and the CDC to gain a better understanding of statewide resistance patterns, rapidly respond to cases of novel or high-concern antimicrobial-resistant organisms, and prevent further spread.

## Materials and methods

An electronic survey was developed in Qualtrics, version March 2024 [5], by the authors based on the isolate submission process to investigate barriers to isolate submission by ACHs and CAHs in Texas. The survey used for this study was modified from a previously published survey by the authors assessing barriers to isolate submission in a smaller region, Texas PHR8 [4]. The authors received approval from DSHS leadership for the modified survey and notified local health departments prior to survey distribution. The survey in its entirety is included in the Appendices. To recruit respondents, regional HAI epidemiologists and local health department epidemiologists distributed the survey via preexisting infection preventionist contact lists maintained at the state and local health department levels.

The survey was distributed via email on March 11, 2024, and was discontinued on April 12, 2024. A total of 211 hospitals were contacted, and three reminders were sent before the survey was discontinued. The survey questions were designed to assess knowledge and awareness of the AR Lab Network throughout Texas and to identify potential barriers to submitting antimicrobial-resistant isolates. Survey responses were analyzed using two-sided Fisher’s exact tests to assess whether variables such as the respondent’s role, length of time in that role, and type of hospital in which they worked were associated with their awareness of AMR and the AR Lab Network and their reported barriers to isolate submission. Each respondent could select multiple barriers to isolate submission, with the potential for many possible combinations that would not yield meaningful interpretations. Therefore, to assess any association between respondent characteristics and perceived barriers to isolate submission, the 12 response options and responses from those who selected "other" were categorized into motivational, resource, or procedural barriers, as displayed in Table 1. Findings were considered statistically significant at a p-value < 0.05. Analyses were performed using RStudio, and a two-sided Fisher’s exact test was applied [6]. 

**Table 1:** Barriers to isolate submission AR Lab Network: Antimicrobial Resistance Laboratory Network.

Motivational	Resources	Procedural
Lack of awareness of the AR Lab Network	Lack of laboratory staff time	Isolates are submitted to a different laboratory
Fear of penalties	Lack of submission training/certified personnel	The submission process is too time-consuming
Lack of incentives	Lack of laboratory and/or shipping supplies	The submission form is too complicated
Lack of interest in participating in the AR Lab Network	Lack of financial ability	

## Results

Of the laboratory personnel in Texas invited to participate, 123 surveys from 211 hospitals were received and analyzed, yielding a response rate of 58.29%; however, only 58 respondents (44.27%) completed the survey in its entirety. Respondents included 21 lab directors (16.03%), 63 laboratory managers/supervisors (48.09%), 11 infection preventionists (IPs) (8.40%), and 14 laboratory testing staff members (10.69%). Thirty-eight (29.00%) respondents indicated that they had worked in their current position for more than 10 years. Of the remaining respondents, 21 (16.03%) had worked in their current position for between five and 10 years, 18 (13.74%) had worked in their current position for three to five years, 39 (29.77%) had worked in their current position for one to three years, and 15 (11.45%) had worked in their current position for less than one year. The respondents’ perception of AMR being a problem at their hospital was not significantly related to either the respondent’s role at the hospital (p = 0.99) or the length of time that the respondent had been working in their current role (p = 0.16) (Table 2). 

**Table 2:** Respondent characteristics and perceived antimicrobial resistance at hospitals Two-sided Fisher's exact test was used to evaluate associations between respondent characteristics and perception of antimicrobial resistance as a problem at the facility where the respondent is employed.

Characteristics	Yes (N = 107 (81.68%))	No (N = 17 (12.98%))	I Don't Know (N = 7 (5.34%))	p-Value
Respondent role				
Infection preventionist (n = 11)	10 (90.91%)	1 (9.09%)	0 (0%)	
Laboratory director (n = 21)	18 (85.71%)	2 (9.52%)	1 (4.76%)	
Laboratory manager/supervisor (n=63)	51 (80.95%)	9 (14.29%)	3 (4.76%)	0.9912
Laboratory testing staff (n=14)	11 (78.57%)	2 (14.29%)	1 (7.14%)	
Other (n=22)	17 (77.27%)	3 (13.64%)	2 (9.09%)	
Duration of employment				
1-3 years (n = 39)	30 (76.92%)	7 (17.95%)	2 (5.13%)	
3-5 years (n = 18)	13 (72.22%)	4 (22.22%)	1 (5.56%)	
5-10 years (n = 21)	17 (80.95%)	4 (19.05%)	0 (0%)	0.1623
Less than 1 year (n = 15)	12 (80.00%)	1 (6.67%)	2 (13.33%)	
More than 10 years (n = 38)	35 (92.11%)	1 (2.63%)	2 (5.26%)	

**Figure 1:**
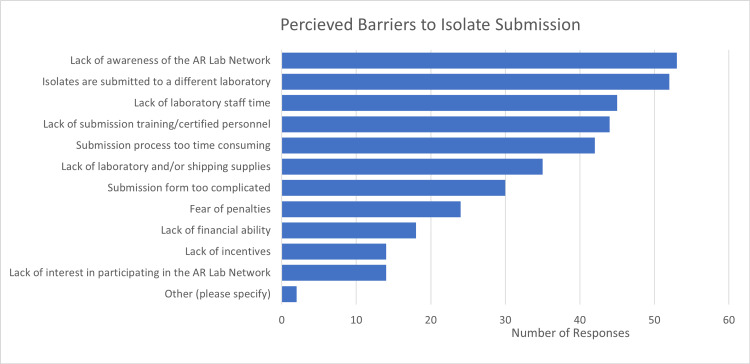
Perceived barriers to isolate submission in Texas Respondents could choose more than one barrier.

Respondents were asked to select up to five choices that they considered the most important barriers to submitting isolates to the AR Lab Network. Lack of awareness of the AR Lab Network was the most frequently cited barrier to submission, with 53 of 105 (50.48%) respondents citing this barrier (Figure 1).

**Figure 2:**
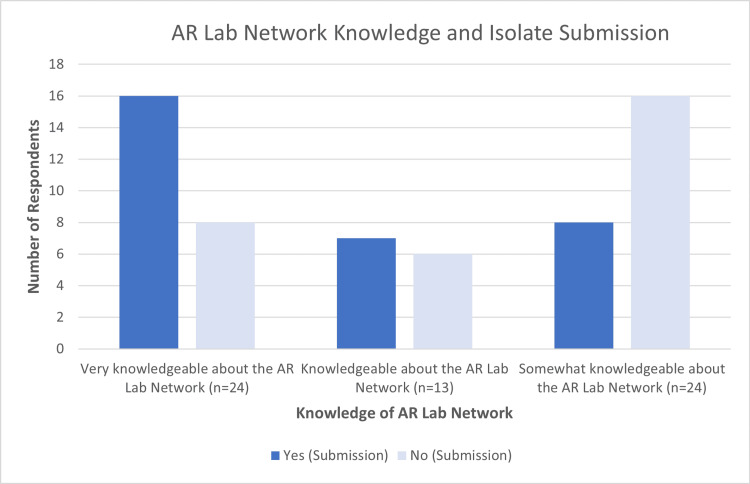
Respondent knowledge of AR Lab Network and submission rate AR Lab Network: Antimicrobial Resistance Laboratory Network.

When rating their own knowledge of the AR Lab Network, 24 (39.34%) respondents indicated that they were “very knowledgeable,” 24 (39.34%) stated that they were “somewhat knowledgeable,” and 13 (21.31%) indicated that they were “knowledgeable.” Only 31 of the 61 individuals(50.81%) who stated that they had heard about the AR Lab Network reported that their hospital actively submits all isolates that meet the AR Lab Network criteria. Neither the facility type nor the level of knowledge reported by the respondent was significantly related to isolate submissions (p = 0.05831) (Table 3 and Figure 2). 

**Table 3:** AR Lab Network knowledge and facility type in relation to submission rate A two-sided Fisher's exact test was used to evaluate the relationship between respondent knowledge of AR Lab Network, facility type, and submission rate. AR Lab Network: Antimicrobial Resistance Laboratory Network.

Characteristics	Yes (N = 31)	No (N = 30)	p-value
Knowledge of AR Lab Network			
Very knowledgeable about the AR Lab Network (n = 24)	16 (66.67%)	8 (33.33%)	0.05831
Knowledgeable about the AR Lab Network (n = 13)	7 (53.85%)	6 (46.15%)	
Somewhat knowledgeable about the AR Lab Network (n = 24)	8 (33.33%)	16 (66.67%)	
Facility type			
Acute care hospital (n=50)	26 (52.00%)	24 (48.00%)	0.749
Other (please specify) (n=11)	5 (45.45%)	6 (54.55%)	

Submission of isolates to a different laboratory was the second most commonly identified barrier to submission, with 52 (49.52%) respondents citing this barrier. Other reported barriers to submission included lack of laboratory staff time, reported by 45 respondents (42.86%); lack of submission training/certified personnel, reported by 44 respondents (41.9%); and the submission process being too time-consuming, reported by 42 respondents (40%) (Figure 1). Using the categories described in Table 1, responses fell into seven combinations of perceived barriers (Tables 4, 5). Neither the respondent’s role at the hospital (laboratory director/manager, IP, or laboratory testing staff) nor the type of hospital in which they worked (ACH or CAH) significantly affected their perceived barriers to antimicrobial-resistant isolate submission (p = 0.67 and p = 0.25, respectively). 

**Table 4:** Respondent role and perceived barriers to AR Lab Network submission Two-sided Fisher's exact test was used to evaluate the relationship between respondent role and perceived barriers to submission of isolates. AR Lab Network: Antimicrobial Resistance Laboratory Network.

Respondent Role	Motivational (N = 7)	Motivational x Procedural (N = 9)	Motivational x Resources (N = 13)	Motivational x Resources x Procedural (N = 42)	Procedural (N = 19)	Resources (N = 5)	Resources x Procedural (N = 19)	p-Value
Infection Preventionist (n = 6)	0	0	2	3	0	0	1	
Laboratory Director (n = 20)	3	1	3	6	4	2	1	0.6719
Laboratory Manager/Supervisor (n = 59)	2	5	6	21	11	3	11	
Laboratory Testing Staff (n = 12)	0	1	0	8	1	0	2	
Other (n = 17)	2	2	2	4	3	0	4	

**Table 5:** Facility type and perceived barriers to AR Lab Network submission A two-sided Fisher's exact test was used to evaluate the relationship between facility type and perceived barriers to isolate submission. AR Lab Network: Antimicrobial Resistance Laboratory Network.

Characteristic	Motivational	Motivational x Procedural	Motivational x Resources	Motivational x Resources x Procedural	Procedural	Resources	Resources x Procedural	p-value
Facility type	N = 7	N = 8	N = 12	N = 40	N = 19	N = 5	N = 19	0.2512
Acute care hospital (n = 78)	6	4	5	29	12	5	17	
Inpatient rehabilitation facility (n = 1)	0	0	0	1	0	0	0	
Long-term acute care hospital (n = 3)	0	0	2	1	0	0	0	
Nursing home/skilled nursing/Assisted living facility (n = 2)	0	0	1	1	0	0	0	
Other (please specify) (n = 26)	1	4	4	8	7	0	2	

One hundred respondents, regardless of their role, reported a willingness to attend a virtual information session about the AR Lab Network (86.21%). When asked how DSHS and the AR Lab Network could encourage facilities to submit isolates, 70 respondents (66.04%) indicated that they would find a hardcopy guide helpful that specified accepted isolate types, shipping steps, and specific tests conducted by the AR Lab Network; 69 respondents (65.09%) stated that providing shipping supplies to the laboratory would increase submissions; and 61 respondents (57.54%) requested provision of virtual or in-person education to facility hospital staff, including IPs, leadership, and other stakeholders. Responses to the question, “Would any of the following strategies be helpful for getting the word out and increasing awareness about the AR Lab Network?” included requests for different types of outreach programs. Of these, 81 respondents (76.42%) requested emailed specimen submission guidance or other educational materials, 50 respondents (47.17%) requested either in-person hospital visits or virtual meetings to introduce the AR Lab Network and explain the isolate submission process, and 12 respondents (11.32%) requested outreach at IP conferences and clinical microbiology conferences. The responses point to several possible ways to overcome barriers to submission of antimicrobial-resistant isolates to the AR Lab Network.

## Discussion

**Figure 3:**
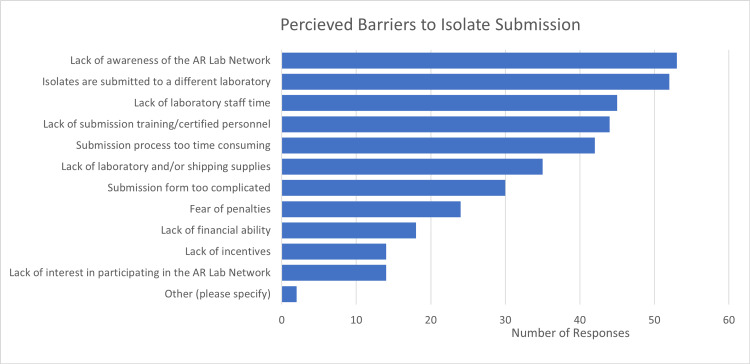
Perceived barriers to isolate submission in Texas PHR8 PHR8: Public Health Region 8.

Consistent with prior work conducted in PHR8 [4], the findings from this statewide survey demonstrate that lack of awareness of the AR Lab Network remains the most significant barrier to isolate submission among hospital laboratories and infection prevention personnel in Texas (Figure 3). 

In the PHR8 analysis, nearly two-thirds of respondents cited insufficient awareness as a primary obstacle, a trend that mirrors the results of the present study and suggests that this challenge persists across geographic regions and hospital types within the state. Similar barriers have also been reported in other jurisdictions, where an incomplete understanding of public health laboratory submission criteria has limited participation in AMR surveillance systems [4, 7].

Survey responses were analyzed to determine whether respondent characteristics (respondent’s role, length of time in that role, and facility type) were associated with awareness of AMR and the AR Lab Network or with reported barriers to isolate submission. Although the majority of respondents across all roles indicated that AMR is addressed as a relevant concern at their facility, no statistically significant association was identified between perceived AMR prioritization and respondent role or experience level, a finding also reported in the PHR8 study. This apparent disconnect may reflect social desirability bias, in which respondents are reluctant to acknowledge gaps in institutional AMR preparedness, particularly in public health surveillance and regulatory oversight. Similar response biases have been documented in infection prevention and laboratory workforce surveys, where perceived expectations influence self-reported practices and attitudes [4, 7].

Notably, 81.68% of respondents reported that AMR is adequately addressed at their facility, which may contribute to complacency and reduced motivation for voluntary submission of isolates. Previous research has demonstrated that when AMR is perceived as a controlled or adequately managed issue, engagement in external surveillance initiatives such as the AR Lab Network may decline, particularly in the absence of mandatory reporting requirements. In contrast, states with mandatory isolate submission policies, such as Michigan, have achieved more comprehensive AMR surveillance coverage, highlighting the impact of regulatory frameworks on submission behaviors [8, 9].

Although not statistically significant, the present study suggests a potential positive relationship between knowledge of the AR Lab Network and isolate submission, consistent with national data indicating that laboratories with greater familiarity with AR Lab Network processes are more likely to participate in submission activities. Importantly, despite widespread lack of awareness, 86.21% of respondents expressed interest in attending a virtual informational session, underscoring the feasibility and potential effectiveness of educational outreach as a low-resource intervention. Educational initiatives targeting laboratory professionals have been shown to significantly improve knowledge of AMR surveillance systems and increase engagement with public health reporting processes [7, 10].

Educational outreach directed at infection preventionists, laboratory directors, and microbiology staff, delivered through virtual or in-person formats, has been identified as a key facilitator of improved AMR surveillance participation across multiple settings. Texas DSHS plans to implement future educational sessions tailored to this audience, with the goal of increasing awareness of the AR Lab Network’s mission, submission criteria, and public health value. Such efforts align with CDC recommendations emphasizing bidirectional communication and sustained engagement between clinical and public health laboratories [7, 11].

In addition to educational interventions, streamlining the isolate submission process may further reduce barriers identified by respondents. Consistent with findings from both this study and prior research, the lack of staff time, training, and shipping resources remain persistent challenge. Providing shipping supplies, clear step-by-step submission guidance, and courier services have been shown in other states to increase submission compliance and reduce operational burden on clinical laboratories. These approaches may be particularly beneficial for smaller facilities and critical access hospitals with limited laboratory infrastructure [4, 12, 13].

Several limitations should be considered when interpreting these findings. The modest sample size and inherent non-response bias associated with survey-based studies may limit generalizability. Of the 123 responses analyzed, only 58 were complete (47.15%) and 65 were incomplete (52.85%). Non-responding facilities may differ systematically from respondents, potentially exhibiting even lower levels of engagement with DSHS and the AR Lab Network. Similar limitations have been noted in comparable AMR surveillance studies and highlight the need for complementary qualitative and administrative data to better characterize non-participating facilities [14].

## Conclusions

This study is novel in its statewide assessment of barriers to antimicrobial-resistant isolate submission across Texas, expanding upon prior regional analyses and providing actionable insights for public health intervention. As in the previous study of PHR8, lack of awareness of the AR Lab Network was the main reported barrier to isolate submission across the state as a whole. The consistency of reported barriers across CAH and ACH suggests that these challenges are not isolated and may extend to other jurisdictions with voluntary submission frameworks. Addressing these barriers through targeted education, logistical support, and sustained collaboration has the potential to significantly enhance antimicrobial-resistant isolate submission rates.

Increased participation in the AR Lab Network will strengthen the ability of Texas DSHS and the CDC to detect emerging resistance patterns, support rapid containment responses, and prevent the spread of high-consequence antimicrobial-resistant organisms. Ultimately, improving isolate submission practices is essential for advancing statewide and national AMR surveillance efforts and protecting public health.

## Appendices

**Table 6:** Survey sent to Texas healthcare facilities AR, antimicrobial resistance; AR Lab Network, Antimicrobial Resistance Laboratory Network; CDC, Centers for Disease Control and Prevention; CRE, carbapenem-resistant Enterobacterales; CRPa, carbapenem-resistant Pseudomonas aeruginosa; CRAb, carbapenem-resistant Acinetobacter baumannii; DSHS, Department of State Health Services. Credit: Rachel Pittman and Rebecca Sanchez.

Question No.	Survey Question	Response Options
—	Facility name	Open response
—	Facility address	Open response
—	Facility county	Open response
—	Facility type	Acute care hospital; Inpatient rehabilitation facility; Long-term acute care hospital; Nursing home/skilled nursing/assisted living facility; Other (specify)
—	Contact information (first and last name, work phone number, email address)	Open response
—	What is your role? (Select all that apply)	Infection Preventionist; Laboratory Director; Laboratory Testing Staff; Laboratory Manager/Supervisor; Other (specify)
—	How long have you been working in your current position?	Less than 1 year, 1-3 years, 3-5 years, 5-10 years, More than 10 years
—	What is the best way to contact you?	Email; Work phone; other (specify)
1	Do you believe antimicrobial resistance (AR) is addressed as a relevant concern at your facility?	Yes; No; I don’t know
1a	Briefly explain the reason for your answer	Open response
2	Do you have knowledge about the AR Lab Network prior to this survey?	Yes; No
2a	If *yes *to Q2: How and/or where have you heard about the AR Lab Network? (Select all that apply)	Texas Department of State Health Services (DSHS) website; American Medical Association (AMA) Antimicrobial Resistance website; Association for Professionals in Infection Control and Epidemiology (APIC) meeting; Conference; Centers for Disease Control and Prevention (CDC) webinar; Communication from health department; Hospital staff/work colleague; Flyer/letter (specify type); Friend; Other (specify)
3	If *yes *to Q2: How would you rate your knowledge of the AR Lab Network?	Somewhat knowledgeable; Knowledgeable; Very knowledgeable
4	Are you willing to attend a virtual informational session about the AR Lab Network?	Yes; No
4a	If *no *to Q4: What alternative types of educational outreach might your facility prefer to receive from the DSHS Laboratory AR Lab Network? (Select all that apply)	Email notifications with AR Lab Network news; Hard copy flyers with specimen collection and shipping guidance; AR Lab network shipping, specimen submission, and supply ordering guidance on DSHS website; Other (specify); I am not interested in receiving educational materials
4b	If *no *to Q4 and not interested in educational materials: Please specify any reason you are not interested in educational outreach	Too much of a time commitment; Information not applicable for our facility; Limited training and staffing capacity; We use other online resources, including CDC ARLN website; We use internal processes for AR surveillance and monitoring; Other (specify)
5	If *yes *to Q4: Does your facility submit isolates to the AR Lab Network at the DSHS Austin Laboratory?	Yes; No; I don’t know
6	If *no *to Q5: Do you know the reason(s) why your facility does not submit AR isolates?	Yes; No; I don’t know
7	If *no *or *I don’t know *to Q6: Would your facility be willing to begin submitting isolates?	Yes; No
8	If *no *to Q7: Please specify the reason(s) why	There is a fear of penalties being imposed on the facility for not submitting isolates to the AR Lab Network; Too much time commitment; Limited training and staffing capacity; We use internal processes for AR surveillance and monitoring; Other (specify)
9	If *yes *or *I don’t know *to Q5: Does your facility submit every AR isolate that meets the AR Lab Network submission criteria?	Yes; No; I don’t know
	Isolates that meet the AR Lab Network submission criteria:	
	Carbapenem-resistant *Enterobacterales *(CRE)	
	Carbapenem-resistant *Pseudomonas aeruginosa *(CRPa)	
	Carbapenem-resistant *Acinetobacter baumannii *(CRAb)	
	Candida auris or an unidentifiable *Candida *species (C.auris)	
	Antimicrobial-Resistant *Neisseria gonorrhoeae* (AR GC)	
10	If *no *or *I don’t know *to Q9: What prompts you to submit AR isolates sometimes? (Select all that apply)	Physician request is required for submission; Health Department (HD) request is required for submission; When we identify unusual resistance (or antimicrobial susceptibility test results) for specific organisms; Other (specify)
10a	If *no *or *I don’t know *to Q9: What isolates does your facility submit sometimes? (Select all that apply)	CRE; CRPa; CRAb; Candida auris; Antimicrobial-resistant *Neisseria gonorrhoeae*; Other (specify)
11	What do you consider the most significant barrier(s) at your facility to submitting isolates that meet AR Lab Network submission criteria? (Select up to five choices)	Lack of awareness of the AR Lab Network; Lack of interest in participating in the AR Lab Network; Lack of training on how to submit isolates/personnel certified to ship infectious substances; Lack of laboratory staff time; Lack of laboratory and/or shipping supplies; Lack of financial ability of submission/shipping; Lack of incentives for submitting isolates; Other (specify)
12	Which of the following statements do you agree with? (Select all that apply)	There is a fear of penalties being imposed on the facility for failing to submit isolates; The process for submitting isolates is too time-consuming; The submission form is too complicated and/or too overwhelming; Isolates are submitted to a different laboratory (list names); All of the above; None of the above
13	What could DSHS Laboratory and the AR Lab Network do to encourage and make it easier for facilities to submit AR isolates? (Select all that apply)	Provide hardcopy guidance/information that identifies the specific tests the AR Lab Network conducts, accepted isolate types, and shipping steps; Provide virtual or in-person specimen submission education to facility staff, including IP, leadership, and other stakeholders; Provide free shipping supplies to your facility; Other (please specify, and provide suggestions, if possible)
14	Which staff member at your facility or organization would be helpful for getting the word out and increasing awareness about the AR Lab Network? (Select all that apply)	Facility Infection Preventionist; Laboratory and/or Microbiology Manager and/or Director; Facility/Organization Chief Executive Officer/Director/Administrator; Other (please specify and list any suggestions you may have)
15	Would any of the following strategies be helpful for getting the word out and increasing awareness about the AR Lab Network? (Select all that apply)	Email with specimen submission guidance or other educational materials; Telephone to provide specimen submission guidance or other educational content; Conduct educational outreach at Infection Prevention and Control and Clinical Microbiology conferences (if selected, please specify conference); Schedule an in-person visit or virtual meeting with your facility to introduce the AR Lab Network and explain the AR isolate submission process; Other (please specify and list any suggestions you may have)

## References

[1] Centers for Disease Control and Prevention (2022). Centers for Disease Control and Prevention (2019). Antibiotic Resistance Threats in the United States.

[2] Texas Department of State Health Services (2022) (2022). Texas Department of State Health Services (2022). Local health department and DSHS regional public health coverage. https://dshs.texas.gov/regions/state.shtm.

[3] Texas Department of State Health Services (2021 (2022). Texas Department of State Health Services (2021). Texas Public Health Region 8. https://www.dshs.texas.gov/region8/overview.shtm.

[4] Moorhead B, Shrestha N, Newman-Caro AB (2024). Determining barriers to submitting antimicrobial-resistant isolates among hospitals in Texas Public Health Region 8. J Infect Prev.

[5] Qualtrics XM (2024). Qualtrics XM. https://www.qualtrics.com.

[6] R Core Team (2019). R: A Language and Environment for Statistical Computing. https://www.R-project.org/.

[7] (2022). Centers for Disease Control and Prevention. Antimicrobial Resistance Laboratory Network (AR Lab Network). https://www.cdc.gov/antimicrobial-resistance-laboratory-networks/php/about/domestic.html.

[8] (2026). Centers for Disease Control and Prevention. AR Lab Network technical appendix. http://arpsp.cdc.gov.

[9] (2026). Michigan Department of Health and Human Services. Antimicrobial resistance reporting in Michigan. https://www.michigan.gov/mdhhs/keep-mi-healthy/infectious-diseases/hai/antimicrobial-stewardship.

[10] Mudenda S, Daka V, Kamayani M (2025). The effect of educational intervention on healthcare workers’ awareness and knowledge of antimicrobial resistance. Pharm Pharmacol.

[11] (2026). Texas Department of State Health Services. Texas AR Lab Network Response Plan. http://dshs.texas.gov.

[12] (2026). Florida Department of Health. 2025 CPO submission criteria. https://www.floridahealth.gov/wp-content/uploads/2026/02/2025-CPO-Submission-Criteria.pdf.

[13] (2026). Montana Department of Public Health and Human Services. Guidance Documents for Sample Submission. https://dphhs.mt.gov/publichealth/LaboratoryServices/GuidanceDocumentsforSampleSubmission.

[14] Akinyele A, Olamijuwon E, Adeniyi AA, Kazeem OS, Popoola M, Agboeze TC, Okeke IN (2025). Barriers and facilitators to digital technology application for antimicrobial resistance surveillance: a co-produced qualitative synthesis. PLOS Glob Public Health.

